# The relation of sarcopenia and disability in multiple sclerosis

**DOI:** 10.1016/j.msard.2023.104855

**Published:** 2023-09

**Authors:** Lukas Haider, Karen K Chung, Stephanie Mangesius, Julia Furtner, Olga Ciccarelli, Declan T Chard, Frederik Barkhof

**Affiliations:** a NMR Research Unit, Queen Square Multiple Sclerosis Centre, University College London Institute of Neurology, United Kingdom; b Department of Biomedical Imaging and Image Guided Therapy, Medical University of Vienna, Austria; c Department of Neuroradiology, Medical University of Innsbruck, Austria; d Neuroimaging Core Facility, Medical University of Innsbruck, Austria; e National Institute for Health Research (NIHR) University College London Hospitals (UCLH) Biomedical Research Centre, United Kingdom; f Centre for Medical Image Computing (CMIC), Department of Medical Physics and Biomedical Engineering, University College London, United Kingdom; g Department of Radiology and Nuclear Medicine, VU University Medical Centre, Amsterdam, NL, USA

**Keywords:** Sarcopenia, Multiple sclerosis, Temporal muscle thickness, Long-term outcome

## Abstract

**Background:**

The relation of sarcopenia and disability in MS is unknown.

**Objective:**

To investigate the relation of temporal muscle thickness (TMT) and disability.

**Methods:**

A cohort of 132 people who presented with a clinically isolated syndrome (CIS) suggestive of MS at a mean age of 30.0 years, were prospectively followed clinically and with MRI over 30-years. TMT and expanded disability status scale (EDSS) were assessed at baseline, one- five- ten- fourteen- twenty- and thirty-year follow-up.

**Results:**

At 30-years, 27 participants remained classified as having had a CIS, 34 converted to relapsing remitting MS, 26 to secondary progressive MS, and 16 had died due to MS.

Using linear mixed effect models with subject nested in time, greater annualized TMT-thinning was seen in individuals who developed MS (-0.04 mm/a, 95%CI: -0.07 to -0.01, *p* = 0.023).

In those who converted to MS, a thinner TMT was reached at 14- (*p* = 0.008), 20- (*p* = 0.002) and 30-years (*p*< 0.001).

TMT was negatively correlated with EDSS at 20-years (*R*=-0.18, *p* = 0.032) and 30-years (R-0.244, *p* = 0.005).

Longitudinally, TMT at earlier timepoints was not predictive for 30-year clinical outcomes.

**Conclusion:**

TMT thinning is accelerated in MS and correlated with disability in later disease stages, but is not predictive of future disability.

## 1 Introduction

Multiple Sclerosis (MS) is a chronic inflammatory neurodegenerative disease of the central nervous system.

In a recent report sarcopenia was detected in one-fifth of MS patients and was associated with a high degree of fatigue and lack of exercise (Yuksel et al., 2021). As with other chronic conditions, there is growing interest in the way in which ageing and disease interact to explain disability. MS affects all aspects of central nervous system functions, including motor, and it is recognised that people with MS develop muscular atrophy, (Bove et al., 2016; Wingo Brooks et al., 2018) potentially due to physical inactivity. However, a smaller skeletal muscle (type I and II) fibre cross sectional area was observed in early stages of the disease (Wens et al., 2014) and altered muscle AMPKα and mTOR signalling (involved in muscular mitochondrial and myofibrillar biogenesis) remained even after activity was restored. (Hansen et al., 2015) It is further unclear, to which extend muscular atrophy is attributable to ageing or MS, and how muscular atrophy is related to present and future disability.

Temporal muscle thickness (TMT) has recently been proposed as a valid surrogate measure for sarcopenia, (Nozoe et al., 2021) that can be easily assessed on brain MRI scans. (Nozoe et al., 2021) TMT is correlated with the volume of other skeletal muscles, (Hasegawa et al., 2019; Leitner et al., 2018) hand grip strength in both healthy controls and people with various neurological diseases, (Steindl et al., 2020) the nutritional status (Hasegawa et al., 2021) and constitutes a well-established prognostic outcome measure in individuals with subarachnoid haemorrhage, (Katsuki et al., 2019) brain metastases, (Furtner et al., 2017; Furtner et al., 2018) glioblastoma (Furtner et al., 2019; An et al., 2020; Yesil and Er, 2020) and primary central nervous system lymphoma. (Furtner et al., 2021)

We recently completed a 30-year longitudinal, clinical and MRI follow-up study of a cohort recruited after a clinically isolated syndrome (CIS) suggestive of MS. (Chung et al., 2020; Haider et al., 2021a, 2021b) In the present study we cross-sectionally and longitudinally assessed the development TMT thinning, and its association with disability as measured by expanded disability status scale (EDSS) scores, measured at baseline, one, five, 10, 14, 20 and 30-year follow-up. We addressed the following questions:

(I) Does TMT differ between people who developed MS and those who remained CIS?
(II) Is TMT cross-sectionally correlated with disability, and if so is this correlation due to age-related sarcopenia, MS-related muscular atrophy, or a combination of them?
(III) Does TMT thinning predict subsequent clinical outcomes?

## 2 Material and methods

### 2.1 Study participants

Clinical (Chung et al., 2020) and radiological (Haider et al., 2021a, 2021b) details of this cohort have been described in detail recently. In short, 132 subjects with a clinically isolated syndrome (CIS) suggestive of MS were prospectively recruited between 1984 and 1987, and underwent clinical assessment and MRI brain scan at baseline, one, five, ten, 14, 20 and 30 years.

Clinical outcomes at 30-years were available in 120 subjects, 13 of whom had died. Of the remaining 107, all had at least one follow-up scan.

Individuals were classified using the 2010 McDonald criteria (Polman et al., 2011).

Clinical details are provided in Table 1, for a more detailed description see Chung et al., 2019. (Chung et al., 2020)

**Table 1:** Study cohort characteristics.

30-year: Classification, MRI		CIS	RRMS	SPMS	MS related death	All
Number		27	34	26	16	103*
Age at CIS [years]		31.7 (8)	29.7 (6.2)	32.1 (7.1)	35.7 (8.6)	31.7 (7.5)
Years CIS to RRMS		NA	6.3 (6.4)	4.5 (5)	2.8 (2.2)	5 (5.4)
Years CIS to SPMS		NA	NA	16.8 (6.6)	12 (4.6)	15.3 (6.4)
Years CIS to death due to MS		NA	NA	NA	21.9 (6.6)	21.9 (6.6)
Age at death [years]		NA	NA	NA	57.6 (11.3)	NA
CIS type	Cord (n)	13	17	16	10	56
	Optic Neuritis (n)	12	12	8	2	34
	Brainstem (n)	2	5	2	4	13
sex	Female (n)	18	22	17	10	67
	Male (n)	9	12	9	6	36
EDSS at 30 years		1 (0 - 2)	1,5 (1 - 2)	6 (6 - 6,5)	NA	3 (1.5–7)

Legend: The number of subjects and clinical core characteristics are provided for each 30-year outcome defined group (adapted from Chung et al., 2019).

Abbreviations: CIS: Clinically Isolated Syndrome; RRMS: Relapsing Remitting Multiple Sclerosis; SPMS: Secondary Progressive Multiple Sclerosis; n: number/count; EDSS: expanded disability status scale.

### 2.2 Ethics

This study was approved by our local ethics committee and the National Research Ethics Service (15/LO/0650). Participants gave informed consent, written if they attended in person, or verbal if they provided clinical information by telephone only.

### 2.3 Image acquisition

**Fig. 1:**
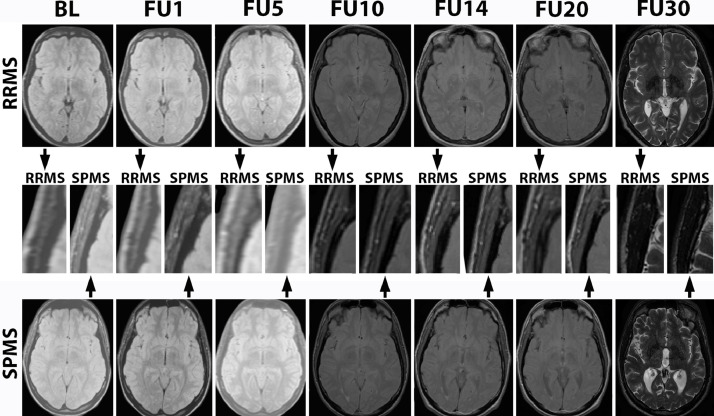
TMT over 30-years Legend: PD/T2 weighted MRIs were acquired at 0/1/5/10/14/20/30-years. The upper row shows a male subject with RRMS out-come (aged 25 at CIS), the lower row a male with SPMS outcome (aged 27 at CIS). The middle row show higher magnification inserts of the right temporal muscle at each follow-up.

Abbreviations: BL: MRI at baseline; FU1/5/10/14/20/30: Follow-up MRI at 1/5/10/14/20/30-years; RRMS: Relapsing Remitting Multiple Sclerosis; SPMS: Secondary Progressive Multiple Sclerosis.

At baseline, 1, and 5 years a Picker 0.5T system (Marconi Medical Systems, Cleveland, OH) was used. At 10, 14, and 20  years, a 1.5T General Electric Signa (GE Healthcare, Chicago, IL) was applied and at 30  years a 3T Philips Achieva (Philips Healthcare, Best, the Netherlands). We acquired Proton‐density and/or T2‐weighted scans at all time points with in-plane resolutions of 1.2 mm, 1 mm and 0.5 mm. Detailed image acquisition parameters are available in **Supplementary Table 1**. Exemplary images are provided in Fig. 1 for each follow-up.

### 2.4 Image analysis

TMT was measured perpendicular to the long axis of the temporal muscle on axial plane at the level of the anterior commissure on PD/T2 weighted images that were available at all follow-up time points (Fig. 1) (LH). 3D reconstructions were not feasible on 2D data. As reported previously, TMT was measured on both sides and is provided as a mean value per patient (Furtner et al., 2017). We did not observe temporal muscle pathologies in our cohort.

### 2.5 Statistical analysis

The statistical analysis was performed with R-studio. (RStudio Team 2020) P-values smaller than 0.05 (two-tailed), were considered statistically significant, and exact values to 0.001 are given.

(I) Does TMT differ between people who developed MS and those who remained CIS?

For cross-sectional group differences in the distribution of TMT at each follow-up time point, subjects were divided into those that remained classified as CIS and those that converted to MS, subsuming RRMS, SPMS, MSRD. Group differences were estimated with linear regression models adjusting for age and sex.

When sarcopenia cut-off values are reported, we use the published sex-adjusted cut-off values defined by 2.5 standard deviations (SD) below the mean TMT values of a normative reference of healthy volunteers between 18 and 40 years at a male cut-off value ≤ 6.3 mm and a female cut-off value ≤ 5.2 mm. (Steindl et al., 2020)

Ad (II) Is TMT cross-sectionally correlated with disability, and if so is this correlation due to age-related sarcopenia, MS-related muscular atrophy, or a combination of them?

Cross-sectional correlations of TMT with EDSS were assessed as non-parametric correlations (Kendall's tau) at each follow-up time point. To account for sex-effects on TMT, semi-partial correlation were applied, where variation from sex is removed only from TMT. (Kim, 2015)

To calculate the differences in the annualized rate of TMT thinning between CIS vs. MS (subsuming RRMS, SPMS, MSRD) we used linear nested mixed effect models. Not all subjects contribute at each time-point and subject ID, as a random factor, was thus nested in time, accounting for differences in time intervals. The models were adjusted for age- and sex-effects.

(III) Does TMT thinning predict subsequent clinical outcomes?

In the longitudinal analysis the relation of TMT at five-years on EDSS at 30-years, as well as reversely the relation of EDSS at five years on TMT at 30-years, was assessed with linear regression models adjusting for age and sex. The beta with a 95% confidence interval and its corresponding p-value is provided.

The likelihood for a subject being characterized as SPMS or MSRD rather than CIS or RRMS based on TMT at five-years was estimated with logistic-regression models adjusting for age and sex. The odds-ratio is provided with a 95% CI and the corresponding p-value.

### 2.6 Data availability statement

Anonymized data, not published in the article, can be shared on reasonable request from a qualified investigator after approval of the local Ethics Committee.

## 3 Results

At 30-years, 27 participants remained classified as having had a CIS, 34 converted to relapsing remitting (RR)MS, 26 to secondary progressive (SP)MS, and 16 had died due to MS(MSRD). Disease duration, age and sex were evenly distributed across the 30-year outcome defined groups. (Chung et al., 2020)

(I) Does TMT differ between people who developed MS and those who remained CIS?

Adjusting for age and gender, significant group differences in the TMT, comparing those who remained classified as CIS, against those who developed RRMS, SPMS or died MS related (MSRD) were present at 14-years (*p* = 0.008), 20-ears (*p* = 0.002) and 30-years (*p*< 0.001). Group differences at earlier follow-ups, 10-years (*p* = 0.102), 5-years (*p* = 0.088), 1-year (*p* = 0.487) and baseline (*p* = 0.158), were not significant (Table 2).

**Table 2:** TMT over time in female and male individuals grouped by 30-year clinical outcome.

	female (*n* = 27)	male (*n* = 76)	Total (*n* = 103)	adj.pvalue
TMT_0				0.158^1^
n-Miss	8	14	22	
Mean (SD)	8.8 (1.6)	8.7 (1.7)	8.7 (1.7)	
Range	5.0 - 13.0	4.7 - 12.0	4.7 - 13.0	
TMT_1				0.478^1^
n-Miss	14	27	41	
Mean (SD)	8.7 (2.1)	8.1 (1.3)	8.2 (1.5)	
Range	5.4 - 12.5	4.7 - 10.6	4.7 - 12.5	
TMT_5				0.088^1^
n-Miss	11	25	36	
Mean (SD)	9.4 (1.3)	8.9 (1.1)	9.0 (1.2)	
Range	7.1 - 11.6	6.6 - 11.5	6.6 - 11.6	
TMT_10				0.102^1^
n-Miss	13	36	49	
Mean (SD)	9.4 (1.0)	8.7 (1.3)	8.8 (1.2)	
Range	7.7 - 11.8	6.1 - 11.3	6.1 - 11.8	
TMT_14				0.008^1^
n-Miss	16	43	59	
Mean (SD)	9.3 (1.1)	8.8 (1.7)	8.9 (1.6)	
Range	6.8 - 11.2	5.1 - 12.7	5.1 - 12.7	
TMT_20				0.002^1^
n-Miss	8	26	34	
Mean (SD)	8.5 (1.0)	7.9 (1.7)	8.1 (1.6)	
Range	6.2 - 10.6	5.2 - 11.9	5.2 - 11.9	
TMT_30				< 0.001^1^
n-Miss	8	34	42	
Mean (SD)	7.4 (1.5)	5.8 (1.9)	6.3 (2.0)	
Range	3.6 - 9.7	2.6 - 10.2	2.6 - 10.2	

Legend: TMT in mm at baseline, 1-, 5-, 10-, 14-, 20-, and 30-year follow-up. P-values result from linear models adjusting for age and sex. Group 0=CIS, Group 1= MS (i.e. RRMS/SPMS/MSRD).

Abbreviations: CIS: Clinically Isolated Syndrome; RRMS: Relapsing Remitting Multiple Sclerosis; SPMS: Secondary Progressive Multiple Sclerosis; MSRD: Multiple Sclerosis Related Death, *N*= number; adj.: adjusted; TMT: Temporal Muscle Thickness.

1 Age and Sex adjusted linear regression model results.

**Fig. 2:**
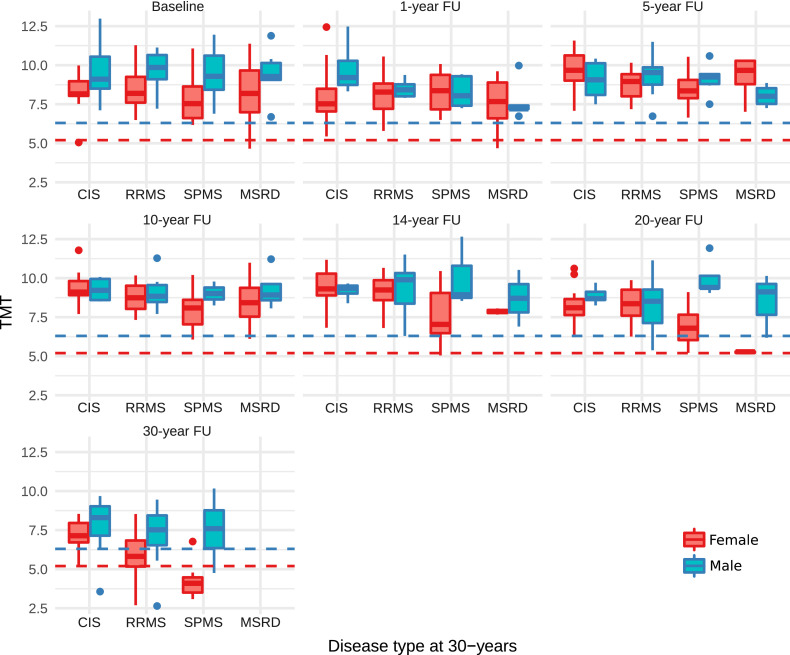
TMT distribution over time in female and male individuals grouped by 30-year clinical outcomes. Legend: TMT in millimetre (mm) at baseline, 1-, 5-, 10-, 14-, 20-, and 30-year follow-up is provided for female (red) and male subjects (blue). Cases are grouped by the 30-year clinical outcome groups CIS, RRMS, SPMS and MSRD. The dashed line indicate published sex specific cut-off values for sarcopenia.

Abbreviations: CIS: Clinically Isolated Syndrome; RRMS: Relapsing Remitting Multiple Sclerosis; SPMS: Secondary Progressive Multiple Sclerosis; MSRD: Multiple Sclerosis Related Death, *N*= number; adj.: adjusted; TMT: Temporal Muscle Thickness.

Sarcopenia, as defined previously with sex-adjusted cut-off values 2.5 SD below the mean TMT values of healthy volunteers aged 18 – 40 (males ≤ 6.3 mm, females: ≤ 5.2 mm, (Steindl et al., 2020)) were primarily reached in at 20, and 30-year follow-ups by female subjects with SPMS (Fig. 2).

(II) Is TMT cross-sectionally correlated with disability, and if so is this correlation due to age-related sarcopenia, MS-related muscular atrophy, or a combination of them?

Accounting for sex-effects on TMT, TMT was cross-sectionally negatively correlated with disability, measured by EDSS, at 30-years (*R*= −0.24, *p* = 0.0059) and 20-years (*R*= −0.18, *p* = 0.0319). The correlations at 14-years (*R*= −0.20, *p* = 0.0581), 10-years (*R*=−0.17, *p* = 0.0731), 5-years (*R*= −0.02, *p* = 0.8158), 1-year (*R* = 0.0, *p* = 0.725) and at baseline (*R*= −0.02, *p* = 0.725) were not significant.

The annualized TMT rate change of the interaction term conversion to MS (subsuming RRMS, SPMS or MSRD out-come at 30-years) and time was −0.04 mm/a; 95%CI: −0.07 to 0.01; p-value= 0.023. Subject age was associated with a lower TMT (−0.04 mm/a; 95% CI: −0.06 to 0.02; p-value <0.001) and the TMT was 0.8 mm thicker in male subjects (95%CI: 0.52 to 1.10; p-value <0.001). Overall this model, including age, sex, follow-up time and the 30-year outcome (CIS vs. MS) explained 75% of the variance in TMT. A closely similar TMT rate was present when the comparison was performed in CIS or RRMS against those that entered the progressive stage of the disease or died due to MS (SPMS + MSRD) (−0.04 mm/a; 95% CI: −0.07 to −0.01; p-value= 0.008) (Table 3).

**Table 3:** Annualized TMT-rate changes.

	CIS vs.MS model		
Predictors	Estimates	CI (95%)	*p*
Intercept (TMT)	10.14	9.17–11.10	<0.001
Age [years]	−0.04	−0.06–−0.02	<0.001
Sex [male]	0.81	0.52–1.09	<0.001
30-year outcome [MS]	−0.21	−0.68–0.27	0.392
time [years]	−0.04	−0.11–0.02	0.152
30-year outcome [MS] * time [years]	−0.04	−0.07–−0.01	0.023
**Random Effects**			
σ2	0.27		
τ00ID	0.55		
τ00time_point	1.35		
NID	100		
Ntime_point	7		
Observations	431		
Marginal R^2^	0.748		

Legend: Nested linear mixed effect regression models were calculated to quantify the annualized atrophy rates in TMT.

Abbreviations: CIS: Clinically Isolated Syndrome; RRMS: Relapsing Remitting Multiple Sclerosis; SPMS: Secondary Progressive Multiple Sclerosis; MSRD: Multiple Sclerosis Related Death, TMT: Temporal Muscle Thickness.

The annualized TMT atrophy rates, for RRMS (−0.16 mm/a; 95%CI: −0.69 to 0.38), SPMS (−0.18 mm/a; 95%CI: −0.74 to 0.39) and MSRD (−0.25 mm/a; 95%CI: −0.91 to 0.41) did not differ from CIS (RRMS: *p* = 0.574, SPMS: *p* = 0.541, MSRD: *p* = 0.462).

(III) Does TMT thinning predict subsequent clinical outcomes?

Adjusting for age and sex, early TMT (TMT at 5-years FU) was not predictive for EDSS at 30-years (*p* = 0.100) (Table 4).

**Table 4:** Prediction of 30-year TMT and EDSS by 5-year data.

	TMT (at 30-years) model			EDSS (at 30-years) model		
Predictors	Estimates	CI (95%)	*p*	Estimates	CI (95%)	*p*
Intercept	5.86	3.49–8.23	<0.001	5.48	−2.74–13.71	0.188
age [years]	−0.05	−0.12–0.02	0.138	0.12	0.01–0.23	0.039
Sex [male]	1.82	0.87–2.78	<0.001	0.33	−1.43–2.09	0.709
EDSS [steps]	−0.45	−0.85–−0.06	0.025			
TMT [mm]				−0.63	−1.38–0.12	0.100
Observations	59			66		
R^2^/R^2^ adjusted	0.293 / 0.254			0.114 / 0.071		

Legend: Linear regression models were calculated to explain TMT at 30-years by EDSS at 5-years and EDSS at 30-years by TMT at 5-years.

The models were adjusted for age and sex.

Abbreviations: EDSS: Expanded disability status scale; TMT: Temporal Muscle Thickness.

Early disability (EDSS at the five-years FU) was predictive for TMT at 30-years. With around −0.5 [mm] in TMT 95%CI:(−0.85 to −0.06) at 30-years, for each EDSS step at 5-years, *p* = 0.025. However, only 29% of TMT at 30-years were explained by this model.

Neither TMT at five-years (*p* = 0.106), nor the change in TMT from baseline to five-years FU data not shown, were predictive for the prediction of CIS vs RRMS/SPMS/MSRD using logistic regression models adjusted for age and sex (Table 5).

**Table 5:** Prediction of 30-year clinical outcome by 5-year TMT.

	RRMS/SPMS/MSRD by TMT at 5-years model		
Predictors	Estimates	CI (95%)	*p*
Intercept (TMT)	62.48	0.23–22,954.44	0.153
Age [years]	1.03	0.95–1.12	0.519
Sex [male]	1.21	0.37–4.15	0.756
TMT at 5-years	0.65	0.37–1.08	0.106
Observations	67		
R^2^Tjur	0.055		

Legend: Logistic regression models were calculated to estimate the effect of TMT at 5-years, and the change in TMT from baseline to five-years on 30-year clinical outcome defined as CIS/RRMS vs. SPMS/MSRD.

The models were adjusted for age and sex.

Abbreviations: CIS: Clinically Isolated Syndrome; RRMS: Relapsing Remitting Multiple Sclerosis; SPMS: Secondary Progressive Multiple Sclerosis; MSRD: Multiple Sclerosis Related Death; TMT: Temporal Muscle Thickness.

## 4 Discussion

Ageing with disease is an area of increasing interest in a variety of conditions, including MS. There is a growing recognition that both age and disease related factors can influence outcomes. Importantly, earlier treatment interventions may delay or prevent disability that would otherwise be precipitated by ageing. In the present study we found evidence for muscular atrophy correlated with disability, attributable to preceding disability and having had MS for over 20 to 30-years.

We observed that time in those that converted to RRMS, SPMS or MSRD, was associated with a greater annualized TMT thinning, suggesting a disease related/ secondary sarcopenia in MS over time. TMT was correlated with disability and significantly thinner in people who accumulated disability towards later follow-up time-points. The concept of secondary sarcopenia in MS was further supported by the longitudinal analysis, as early disability was predictive for a thinner TMT after 30-years. However, TMT at five years was not predictive for final out-come.

Clinically relevant exercise programs exist for individuals with MS. (Kjolhede et al., 2015) As TMT, is readily available on MRI scans performed in the course of clinical routine follow-up and easily applicable, our data suggest that it could guide the early identification of subjects that would thus benefit most from dedicated training and life-style interventions, in order to avoid the vicious circle of immobility. (Becker and Stuifbergen, 2004; Asano et al., 2013)

While 30 years of MRI data provide a uniquely long-term view on the evolution of CIS and MS, there have been technical changes that will have affected the performed measurements. A Picker 0.5T system was used at baseline, 1 and 5 years, 1.5T General Electric Signa at 10, 14, and 20 years and 3T Philips Achieva at 30 years. While in-plane resolutions (in which the measurements were performed) were comparable over time (Fig. 1, **Supplementary Table 1**) rates of change calculated using each of these scanners should be considered with caution based on this consideration. Furthermore, TMT in previous studies was measured on axial T1 weighted images orientated along the anterior - posterior commissure. (Furtner et al., 2017) As isotropic data were not available, we measured at this level, but could not perfectly re-align within the axial plane. However, both absolute values and rate changes are comparable to previous literature. (Steindl et al., 2020; Cruz-Jentoft et al., 2019) While TMT as a surrogate marker for sarcopenia only reflects aspects of more complex regional muscular atrophy patterns, sarcopenic cut-off values were reached in our cohort at a comparable frequency as in previous recent literature using more extensive sarcopenia measures. (Yuksel et al., 2021) We therefore consider the presented data sufficient to support the biological concept of accelerated, disability correlated sarcopenia in MS, but can not provide cut-off values to guide current clinical decision making based on archival film material.

As healthy control data were not acquired when this study was initiated in the early 1980′s, we can only compare against those who remained classified as CIS. We do not think that this fundamentally undermines our main finding that, without treatment, following a CIS relatively more temporal muscle atrophy over time is present in those that developed MS or died in the course of the disease.

In line with previous literature we report the mean TMT value based on measurements from both sides, this might be especially important in MS as muscular asymmetry has been reported, e.g. in the musculature of the lower extremities in individuals with MS (Larson et al., 2013) with compensatory changes in the transversus abdominis -, quadratus lumborum -, and the low-back extensor muscle group. (Ketelhut et al., 2015) Overall the inherent limitations potentially reduced our sensitivity to detect associations or group differences but did not introduce systematic bias.

**Fig. 3:**
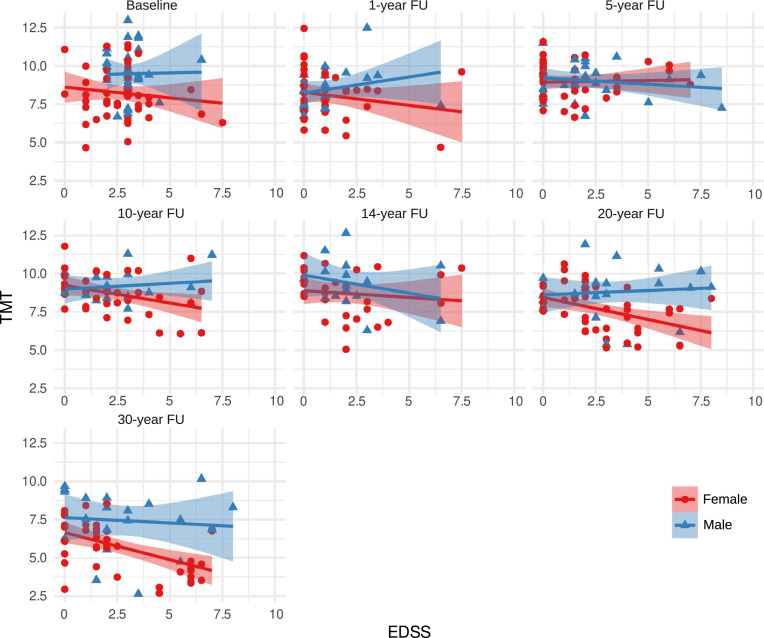
Correlation of TMT and EDSS over time in female and male individuals Legend: TMT in mm at baseline, 1-, 5-, 10-, 14-, 20-, and 30-year follow-up is provided for female (red) and male subjects (blue) plotted against corresponding EDSS values. The regression line is provided with a 95% CI, along with Pearson correlation coefficients and p-values.

Abbreviations: R: Pearson correlation coefficient; TMT: Temporal Muscle Thickness

In conclusion TMT thinning is attributable to ageing and is accelerated over time in individuals with MS, cross-sectionally correlated with disability in later disease stages, but is not predictive of future disability (Fig. 3).

## Declaration of Competing Interest

LH has no conflicts of interest relevant to this study.

KC has received honoraria for speaking at meetings, advisory work or support to attend meetings from Merck, Biogen Idec, Sanofi Genzyme and Roche.

SM has no conflicts of interest relevant to this study.

JF has no conflicts of interest relevant to this study.

OC has served as a consultant for Novartis and has received a speaker honorarium from Merck. She has obtained funding from NIHR, UCLH NIHR BRC, National and UK MS Society, PMSA, and MRC.

DC is a consultant Hoffmann-La Roche. In the last three years he has been a consultant for Biogen, has received research funding from Hoffmann-La Roche, the International Progressive MS Alliance, the MS Society, and the National Institute for Health Research (NIHR) University College London Hospitals (UCLH) Biomedical Research Centre, and a speaker's honorarium from Novartis. He co-supervises a clinical fellowship at the National Hospital for Neurology and Neurosurgery, London, which is supported by 10.13039/100004334Merck.

FB is a consultant for Biogen, Bayer, Merck, Roche, Novartis, IXICO and Combinostics; has received funding from European Commission Horizon (2020), UK MS Society, National Institute for Health Research University College London Hospitals Biomedical Research Centre and GE healthcare; and serves on the editorial boards of Radiology, Brain, Neuroradiology, Multiple Sclerosis Journal, and Neurology.

## Appendix Supplementary materials

Image, application 1
