# Supplementary material for: The relation of sarcopenia and disability in multiple sclerosis
Source: Mult Scler Relat Disord. 2023 Sep;77:None. doi: 10.1016/j.msard.2023.104855 (PMC11752834; doi:10.1016/j.msard.2023.104855)
Supplement: Supplementary file 1 [file mmc1.docx]

**Supplementary**

**Table 1**

|  | Follow-up (years) | | | | | | |
| --- | --- | --- | --- | --- | --- | --- | --- |
|  | 0 | 1 | 5 | 10 | 14 | 20 | 30 |
| Manufacturer and field strength | Picker 0.5T | Picker 0.5T | Picker 0.5T | GE Sigma  1.5T | GE Sigma  1.5T | GE Sigma  1.5T | Philips Achieva 3T |
| Axial slice thickness (mm) | 5 or 10 | 5 or 10 | 5 | 5 | 5 | 5 | 3 |
| Axial in plane resolution (mm x mm) | 1.2 x 1.2 | 1.2 x 1.2 | 1.2 x 1.2 | 1.0 x 1.0 | 1.0 x 1.0 | 1.0 x 1.0 | 0.5 x 0.5 |
| Axial TE (ms)  (PD/T2-weighted) | 60 | 60 | 60 | 30/90 | 14/98 | 17/102 | 85 |
| Axial TR (ms)  (PD/T2-weighted) | 2000 | 2000 | 2000 | 2000 | 2000 | 2000 | 4375 |

| Title: | MRI acquisition parameters |
| --- | --- |
| Legend: | The number of subjects and clinical core characteristics are provided for each 30-year outcome defined group. |
| Abbreviations: | T: Tesla; TE: Time to Echo; TR: Time to Repetition; PD: Proton Densitiy; mm: millimetre; ms: millisecond |
